# Magnetic Resonance Imaging in Amyotrophic Lateral Sclerosis

**DOI:** 10.1155/2012/608501

**Published:** 2012-07-09

**Authors:** Katja Kollewe, Sonja Körner, Reinhard Dengler, Susanne Petri, Bahram Mohammadi

**Affiliations:** ^1^Department of Neurology, Hannover Medical School, 30625 Hannover, Germany; ^2^Department of Neurology, International Neuroscience Institute (INI), 30625 Hannover, Germany

## Abstract

Amyotrophic lateral sclerosis (ALS) is a rapidly progressing neurodegenerative disorder which is incurable to date. As there are many ongoing studies with therapeutic candidates, it is of major interest to develop biomarkers not only to facilitate early diagnosis but also as a monitoring tool to predict disease progression and to enable correct randomization of patients in clinical trials. Magnetic resonance imaging (MRI) has made substantial progress over the last three decades and is a practical, noninvasive method to gain insights into the pathology of the disease. Disease-specific MRI changes therefore represent potential biomarkers for ALS. In this paper we give an overview of structural and functional MRI alterations in ALS with the focus on task-free resting-state investigations to detect cortical network failures.

## 1. Introduction

Amyotrophic lateral sclerosis (ALS) is a devastating neurodegenerative disease which affects not only motor function but also involves extramotor systems. According to the revised El Escorial criteria for the diagnosis of ALS the presence of signs for the affection of both upper motor neurons (UMN) in the primary motor cortex and lower motor neurons (LMN) in brain stem and spinal cord is mandatory, and the disease must be progressive [1]. ALS, has a wide variety of clinical phenotypes, and it is therefore sometimes difficult to differentiate ALS from other ALS-mimicking conditions. For the detection of LMN involvement in different body regions, electromyography (EMG) can be used in addition to the clinical examination. UMN signs, on the other hand, must be visible at the clinical examination while electrophysiological transcranial motor stimulation (TMS) abnormalities are not accepted for the diagnosis of ALS according to the El Escorial criteria. Therefore it would be very advantageous to have an additional technical method which sensitively monitors UMN involvement. Magnetic resonance imaging (MRI), and here in particular diffusion tensor imaging (DTI), represents a promising technique for early detection of alterations in the motor cortex and pyramidal tracts. Different other MRI techniques are also currently being developed to serve as biomarkers for earlier and more accurate diagnosis of ALS. A biomarker for UMN affection would further be useful to monitor the neurodegenerative process and therefore disease progression, that is, within clinical trials. Guidelines for the use of neuroimaging in the management of motor neuron diseases have recently been published by the European Federation of Neurological Societies (EFNS) [2]. In 2010, an international group of experts has met at Oxford University, UK, to define essential parameters for future research needed to promote MRI as a biomarker for ALS.

It was concordantly proposed to initiate longitudinal and multicenter studies and thus to analyze larger sample sizes so that results can be optimized and MRI can become a more specific diagnostic tool. Within MRI, one must distinguish between structural and functional MRI techniques. Structural MRI detects morphological changes in grey and white matter. The diffusion tensor imaging method can be used for tractography (e.g., imaging of pyramidal tracts) or to study the connection between different cortical grey matter areas. Structural MRI at present mainly serves to rule out other diseases mimicking ALS but is also supposed to be useful in finding cortical atrophy in ALS. Functional MRI (fMRI) can detect cortical activations corresponding to a task (e.g., motor task) performed by the participant during scanning. The resting-state technique detects fluctuations in different cortical areas during rest (no task performance needed) and visualizes different functional networks such as the sensori-motor network, visual network, and others. FMRI methods are therefore capable of detecting ALS-related differences in brain activation, compensation, and reorganisation. 

This paper describes the structural and functional MRI alterations which have been found in ALS to date, with a particular focus on task-free resting-state investigations to detect cortical network failures.

## 2. Structural Magnetic Resonance Imaging

### 2.1. Voxel-Based Morphometry

The voxel-based morphometry (VBM) technique can be used for the analysis of volumetric changes in gray or white matter (GM; WM) in the brain [3–5]. It is an automated analysis of changes in brain volume using high-resolution three-dimensional T1-weighted MRI scans. During the statistical process, potential structural changes in individual patients are compared to a template of age-matched controls. By this approach, neuroanatomical differences can be detected with much greater sensitivity [2, 6–8].

#### 2.1.1. Motor Cortex

**Figure 1:**
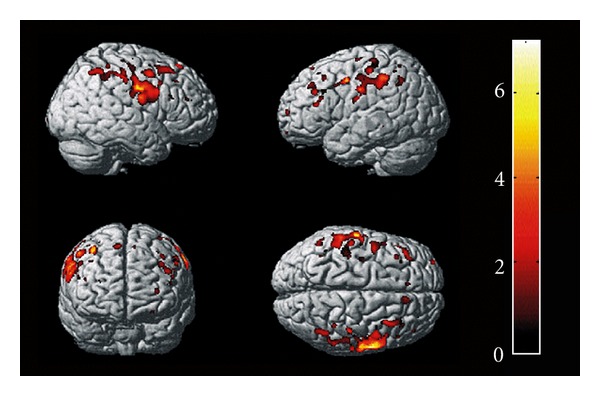
Regional grey matter atrophy in ALS patients compared with controls: group comparison of ALS patients versus healthy controls showed regional grey matter atrophy in the precentral and postcentral gyrus bilaterally, which extended from the primary motor cortex to premotor, parietal and frontal regions bilaterally. The colour bar indicates the statistical strength of the regional atrophy (yellow-white is most significant). Adapted from Grosskreutz and coworkers [4].

Several VBM studies have described atrophy of the primary motor cortex (Figure 1) but this has surprisingly not been a consistent finding in all studies published so far [3, 4, 9, 10]. Marked decreases in the grey matter in the bilateral paracentral lobule were also detected, indicating that the premotor cortex is also involved in degenerative processes in ALS [11]. 

#### 2.1.2. Extra-Motor Involvement

Regional gray matter loss measured by VBM extends to the frontal, temporal, parietal, occipital and limbic regions of the brain and has also been described for the corpus callosum and the cerebellum which is line with clinical and neuroanatomical data [3, 5, 10, 12–14].

## 3. Diffusion Tensor Imaging (DTI)

It is known from postmortem studies in ALS brain specimens that there are extensive white matter abnormalities in the region of the central sulcus and the corticospinal tract (CST), extending across the corpus callosum and into the frontal lobes [15, 16].

To investigate white matter and the directionality of fiber tracts, diffusion tensor imaging (DTI) detects alterations in the degree (axial diffusivity, AD) and directedness (fractional anisotropy, FA) of proton movement. It is sensitive to the direction of water movement *in vivo.* As the diffusion properties of water molecules, demonstrated by DTI, are restricted by the presence of barriers (e.g., cellular membranes), the water molecules tend to diffuse preferentially in orientations along axons, leading to an anisotropic diffusion. Therefore, DTI is used to detect pathology within neuronal white matter tracts and reflects microstructural tissue changes [13]. FA is reduced with loss of neuronal pathway integrity; mean diffusivity (MD) is increased with a loss of neuronal pathway integrity [7].

### 3.1. Corticospinal Tract (CST)

The CST is the structure most frequently studied by DTI in ALS [3, 4, 17] and decreased fractional anisotropy (FA) values in this area have consistently been reported [18–22]. To date, correlation of disease severity and decreased FA has been controversially debated, as some studies found a relation between these factors [20, 23, 24] whereas others did not [25–27]. It is also discussed controversially if increased mean diffusivity (MD) correlates with disease duration, as this was reported by [24, 26, 28], but not by other authors [23]. One other group demonstrated a correlation of a lower mean FA in the CST with rapid disease progression [29].

### 3.2. Corpus Callosum (CC)

Neuropathological studies have shown involvement of the corpus callosum (CC) in ALS [15, 16] and so did several DTI studies, which observed FA changes within the CC of ALS patients [30–36]. The largest FA changes were observed in the posterocentral portion of the CC which is known to link the two motor cortices [9, 16]. The involvement of the CC at an early disease stage would be in line with recent clinical studies [37, 38] and provide an explanation for the focal onset followed by a rapidly spreading progression of the disease.

Unfortunately, the changes in the CC are not specific and were also found in patients with other diseases of the upper motor neuron such as hereditary spastic paraparesis [39, 40] while not detectable in a lower motor neuron syndrome as Kennedy's disease [41].

### 3.3. Extramotor Involvement

FA was shown to be decreased in the premotor white matter (WM), in the prefrontal white matter, and in the temporal white matter [11, 28, 30, 34–36].

### 3.4. Spinal Cord

The small diameter of the spinal cord and its surroundings and breathing-mediated movement artefacts make it difficult to investigate the spinal cord by DTI [42]. In one study, the cervical cord has been investigated and compared to controls; ALS patients showed significantly lower FA of the cervical cord while MD did not differ between the two groups [18]. But during the course of the disease (9 months followup) [43], FA showed a significant decrease and MD showed a significant increase in the spinal cord of ALS patients. A further study supports the hypothesis that the degenerative process in ALS is mostly a “dying-back” mechanism, as the distal part of the spinal cord was the most altered one [7, 44].

### 3.5. Summary I (Structural MRI)

**Figure 2:**
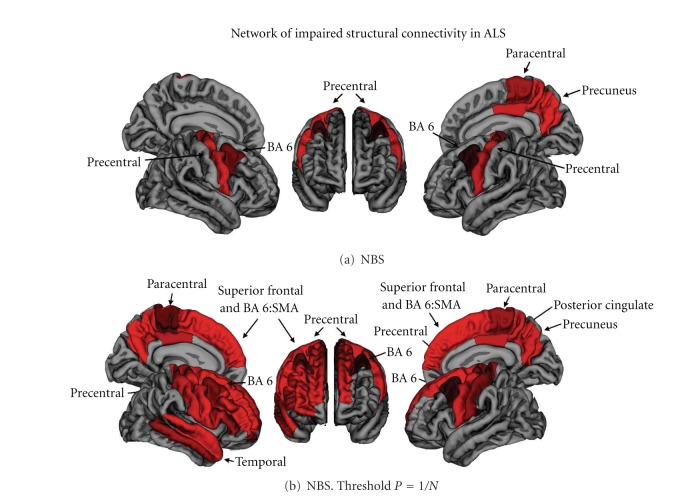
Cortical brain regions with impaired structural connectivity in ALS patients. (a) The network-based statistic procedure revealed a subnetwork of brain regions showing significantly reduced structural connectivity in ALS patients, compared to healthy controls. (b) Using an NBS threshold of *P* = 1/*N* (*N* being the number of nodes of the network) a similar but more extended network was revealed. The model-free approach revealed a sub-network consistent with known motor regions, including precentral and paracentral gyri (primary motor), and caudal middle frontal and superior frontal gyri (supplemental motor areas, BA 6). Adapted from Verstraete and coworkers [46].

According to the consensus guidelines on MRI protocols for studies in ALS patients, DTI is the most promising structural MRI method to detect ALS-related changes not only in the primary motor cortex and the pyramidal tracts but also in brain regions beyond the motor system. DTI scans with a minimum of 12 gradient directions (isotropic voxels with a maximum of 2.5 mm slice thickness) have previously been recommended [42], although, especially for longitudinal studies, 20–30 directions would be preferable in order to permit robust diffusivity measurements [45]. Studies in larger patient cohorts and repeated measurements in the same patients throughout disease progression are necessary to develop DTI as a potential biomarker for preclinical UMN involvement or as a tool to monitor disease progression and the response to therapy in ALS. Beside FA and MD, measuring the strength of connectivity between different anatomical clusters of grey matter can reveal alterations in cortical networks in ALS patients compared to healthy controls. Using DTI one can calculate the connectivity between cortical areas as shown in the following figure (Figure 2, [46]). This novel approach may contribute to an increase in sensitivity and specificity of DTI in ALS.

## 4. Functional Magnetic Resonance Imaging

Functional MRI (fMRI) means the visualization of brain regions in action and is typically done using BOLD-weighted MRI.

BOLD—fMRI takes advantages of the oxygenation level of blood, which is different during rest and activity of the brain when the brain is active, despite the increase in oxygen consumption, there is a subsequent increase in local blood flow that paradoxically results in a decrease of concentration of deoxygenated haemoglobin in the local microvasculature of the activated region. Oxygenated hemoglobin is weakly diamagnetic, while deoxygenated hemoglobin is strongly paramagnetic, thus an increase in the relative concentration of oxygenated hemoglobin results in a lengthened T2*, giving an increase in local MRI signal for T2*-weighted MRI.

This change leads to an increase in the fMRI signal approximately 4 seconds after the neural event in the brain. Thereafter, an equilibration of oxy- and deoxyhemoglobin succeeds the “deactivation phase.” This contrast alone is too weak to show differences to the surrounding brain regions. Comparisons with the same region at rest have to be done followed by special analysis methods [6, 47–49]. The advantages of the BOLD technique are evident: it is noninvasive, provides high resolution, and has a wide accessibility.

Studies with fMRI using a motor task have shown increased cortical activity in ALS patients in the ipsi- and contralateral sensorimotor cortex, supplementary motor area, basal ganglia, and cerebellum [50–53]. This has been discussed as being either the result of cortical adaptation due to peripheral weakness [52] or of cortical reorganisation [50]. In a recent study we have demonstrated that increased cortical activation can be detected even when the performing hand was clinically not affected and interpreted this as a sign of cortical reorganisation in clinically early stages of disease. In this study we could show that early and late phases of neuroplastic changes in ALS can be distinguished according to different disease stages [54]. In another fMRI study we have described for the first time that the pattern of cortical activation during tongue movements differs in ALS patients with and without bulbar signs [55]. We have further investigated this finding by repeated measurements during disease progression in ALS patients with limb and/or bulbar signs, using two different motor tasks (vertical tongue movement and movement of the right hand). In this study, we detected two different patterns of cortical activation changes which were dependent on the presence or absence of bulbar signs. This observation suggests fundamental differences in the neurodegenerative process and subsequent reorganisation mechanisms according to the affected body regions, which apparently can exist in parallel in the same patients [56].

As it is difficult to control task performance in patients with motor deficits, the analysis of “functional connectivity” of spatially remote brain regions has recently gained increasing interest in neuroimaging research in ALS. The idea is that during rest spontaneous coherent fluctuations of the BOLD signal exist in different brain areas which are functionally connected [57]. Resting-state imaging of discrete cortical networks provides a new technique to explore ALS as system failure of interconnected networks [42]. This method only take minutes to acquire and does not suffer from performance confounds that may be present in patients with cognitive or motor impairments [58–60]. It is therefore more suitable for clinical use and in particular for multicentre studies.

**Figure 3:**
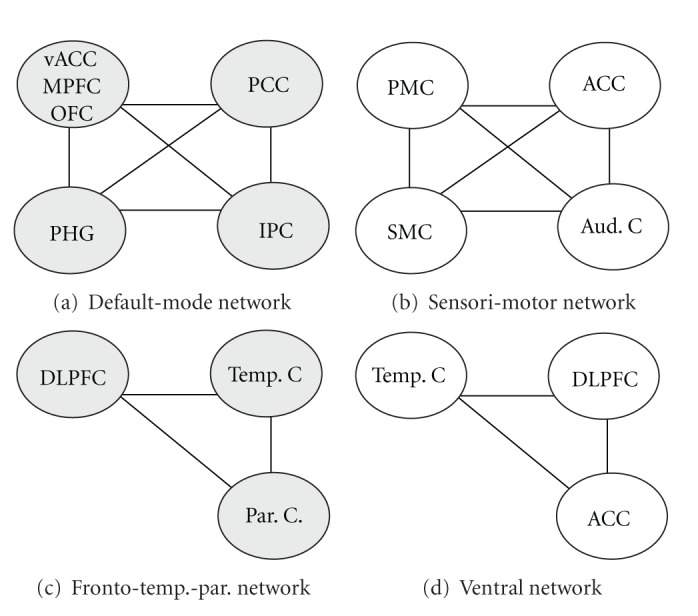
Schematic presentation of 4 reliable recovered networks, adapted from Kollewe and coworkers [69]. (a) *Default*-*mode network*: this network has been reviewed by Raichle and Snyder [65] who have described that activity in this network is high during rest and reduced during cognitive activity. It comprises a large frontal area, including ventral anterior cingulated cortex (vACC), medial prefrontal cortex (MPFC) and orbitofrontal cortex (OFC), the posterior cingulated cortex (PCC), the inferior parietal cortex (IPC), and a temporal region involving the parahippocampal gyrus (PHG). (b) *Sensori-motor network*: this network has been previously identified by a number of authors [62, 66–68]. This network comprises the primary motor cortex (PMC), premotor cortex (PMC), anterior section of cingulated cortex (ACC), the somatosensory region (SSC), and auditory cortex (Aud. C). (c) *Fronto-temporo-parietal network*: this network includes prefrontal (BA9, BA10, BA11), temporal (BA20, BA27), and parietal (BA7, BA39, BA40) regions. (d) *Ventral network*: this network comprises the middle temporal gyrus (Temp. C, BA21), parts of the frontal cortex (DLPFC, BA9, BA47), and parts of the cingulated gyrus (ACC, BA31, BA24). The *posterior network *is not shown; it comprises mainly visual areas in the occipital cortex including BA18 and BA19.

There are different typical resting-state networks which can be recovered from the BOLD signal with high reliability across individuals and studies (Figure 3) [58, 61–63]. One of the consistently recovered networks is the default-mode network (DMN) which is conceptualized as a stand alone cognitive network [64, 65]. It comprises a large frontal area including the ventral anterior cingulate cortex (vACC), the medial prefrontal cortex (MPFC) and the orbitofrontal cortex (OFC)), the posterior cingulate cortex (PCC), the inferior parietal cortex (IPC), and one temporal region, the parahippocampal gyrus (PHG) [62, 66, 67]. Another often reported network is the sensorimotor network [58, 61, 62] which includes the primary motor cortex (PMC), the anterior part of the cingulate cortex (ACC), the somatosensory region (SSC), and the auditory cortex (Aud. C) [62, 66–68]. In addition, several other networks such as a visual executive network have been described [58, 61, 62].

ALS is a neurodegenerative disease which involves mainly the motor system, but already early descriptions [70] and more recent neuropsychological [71–74], electrophysiological [75–77], neuropathological [14], and neuroimaging [78–80] studies pointed out that other than the motor regions of the nervous system are involved in the degenerative process. 

We analyzed for the first time the resting-state networks in ALS patients [57]. Given the definition of ALS as a motor neuron disease, we expected most prominent differences between ALS patients and healthy controls in the sensori-motor network. In view of the increasing knowledge about extramotor involvement in ALS as described above, we also suspected differences between ALS patients and healthy controls in the default-mode network.

We investigated 20 patients suffering from ALS and 20 healthy age-matched controls in a 3-Tesla Siemens Magnetom Allegra Scanner (Erlangen, Germany). The first group consisted of 20 patients, who fulfilled the diagnostic criteria for probable or definite ALS during the course of the disease according to the revised El Escorial criteria of the World Federation of Neurology [1]. The control group comprised 20 healthy volunteers. During the data acquisition for functional connectivity the subjects were instructed to neither engage in cognitive nor motor activity. Analysis and visualization of the data were performed using BrainVoyager QX (Brain Innovation BV, Maastricht, The Netherlands) software.

Applying independent component analysis (ICA), different robustly reproducible functional networks could be extracted from the resting state in both groups [81–85]. Only in two networks, the default-mode and the sensori-motor networks, we found significant differences between ALS patients and healthy controls.

### 4.1. Default-Mode Network (DMN)

**Figure 4:**
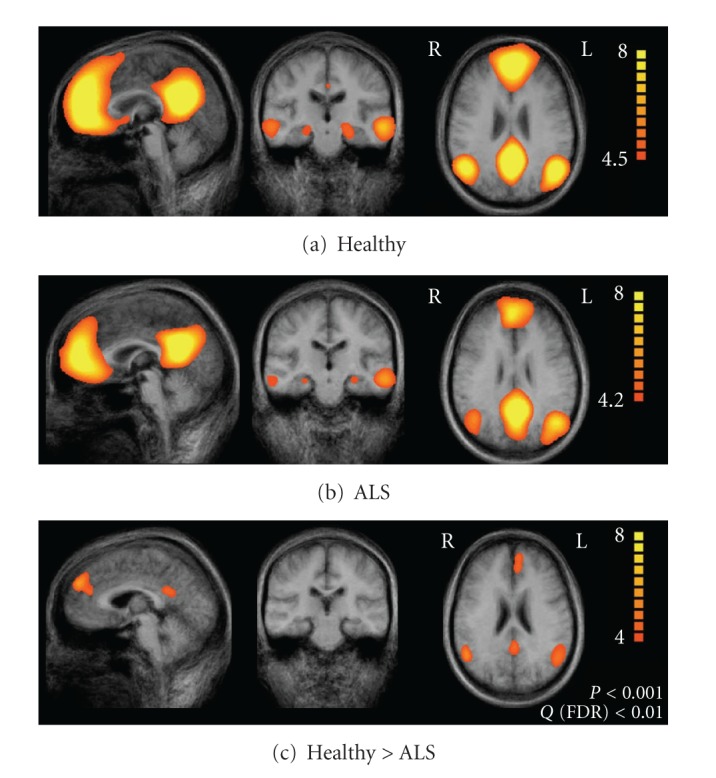
Default-mode network. Upper row (a) illustrates the result of the group ICA analysis for the healthy control participants. The middle row (b) illustrates the results for the ALS patients. The statistical comparison is shown in the lower row (c). Adapted from Mohammadi and coworkers [57].

This network has received considerable attention over the past few years. In the study presented here, we found distinct differences of the default-mode network comparing healthy subjects with ALS patients; in ALS patients we found a significantly decreased connectivity in the lateral prefrontal cortex (BA9), PCC (BA 23), and IPC (BA39) (Figure 4). The PCC, MPFC, and the bilateral IPC are seen as “core hub” of this network and showed a strong intraregional correlation with each other and a weaker correlation with the remaining regions such as the temporal cortex and the medial temporal lobe [57, 86]. Considering our data, functional connectivity is decreased in the core hub of the default-mode network in ALS patients without affecting subcortical (PHG) or temporal regions. In the prefrontal region we found decreased connectivity in BA 9 which is typically involved in working memory tasks, in tasks of sustained attention, and (bilaterally) in tasks demanding problem solving [87]. In IPC we found reduced connectivity in ALS patients located in BA 39. Left BA 39 is known to be involved in perception, recognition, and recall of written language as well as in problem solving [88]. 

All in all, the particular pattern of differences between ALS and control subjects for the default mode network (DMN) bodes well with previous neuropsychological studies suggesting an impairment of higher level executive functions [72–74, 89–91].

### 4.2. Sensori-Motor Network

**Figure 5:**
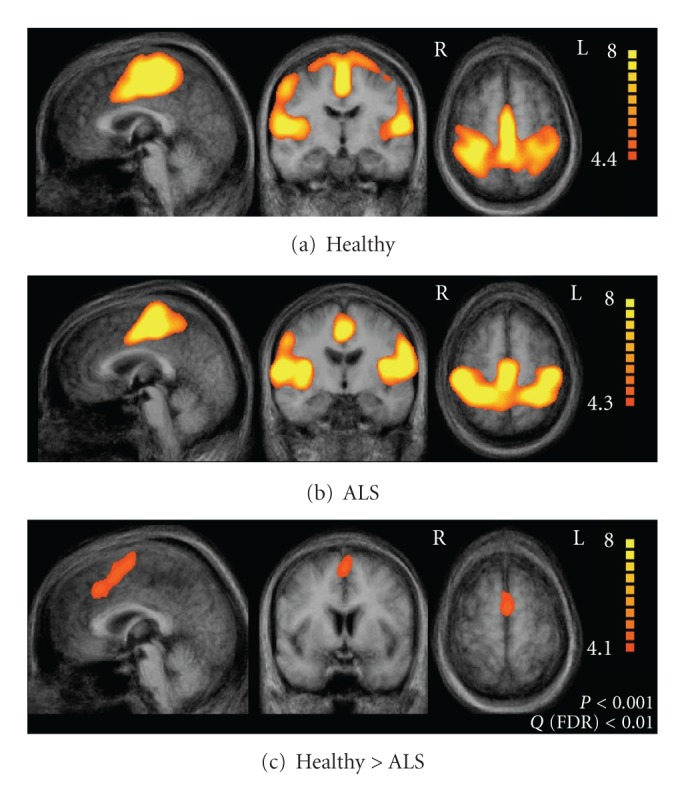
Sensori-motor network. Upper row (a) illustrates the result of the group ICA analysis for the healthy control participants, the middle row (b) illustrates the results for the ALS patients. The statistical comparison is shown in the lower row (c). Adapted from Mohammadi and co-workers [57].

Regarding the sensori-motor network, our study detected differences between ALS patients and controls only in the premotor area (BA6) (Figure 5). All other regions of the sensori-motor network, in particular the precentral gyrus, did not show significant differences between the two groups. Alterations in premotor cortex activity have been demonstrated in a number of functional imaging studies in ALS [50–53, 92, 93] but it has been discussed that these changes might be due to the fact that the same task might be more difficult for ALS patients (and hence associated with increased activation) rather than due to genuine functional changes. As in the present approach no task is imposed on the subject, our data favour a primary functional involvement of the premotor cortex in ALS.

Taken together, we demonstrated significant changes in the DMN and the sensori-motor network. This suggests a disease-specific alteration of these two networks. The DMN has been linked to cognitive processes whereas the latter has been shown to be involved in motor control. The present results once again support extra-motor involvement in ALS.

In the mean time, several further studies have addressed the issue of functional connectivity in ALS by analysis of resting-state data, with partially conflicting results. In a study in 20 ALS patients and healthy age-matched controls, alterations in sensori-motor network in the ALS patient group were detected, similar to our data, but significant changes were seen only in a subgroup of ALS-patients in the DMN, and in the right frontoparietal network [94].

In another study, 25 ALS-patients and age-matched healthy controls were investigated using a combined method to study both structural and functional connectivity [95]. This integrated approach identified apparently dichotomous processes characterizing the cerebral network failure in ALS with increased functional connectivity within regions of decreased structural connectivity. This may point out an interaction between functional and structural connectivity. The atrophy of white matter between distinct cortical areas (reduced structural connectivity) correlates to a higher synchronization of BOLD and an increased interplay between these areas (functional connectivity).

In a third study by the so-called “seed-based analysis” no significant changes in the functional connectivity of the motor system were seen [96]. By this method one searches for a simple correlation between two predefined areas. These partially controversial results of the different studies which investigated resting-state network changes in ALS might be due to methodological differences or to different compositions of the patient groups with different grades of disease severity and therefore different pathological stages. As recently recommended [42], further studies with greater numbers of patients including sufficient numbers of patients in different disease stages could provide better insight into changes of the distinct cerebral networks and their relation to the disease process. In the future, it will be important to pursue multimodal approaches looking for grey matter changes, structural connectivity and functional connectivity, and their correlation with different clinical scores (ALSFRS, neuropsychological parameters, motor performance).

## 5. Conclusion

Of the currently available structural and functional MRI techniques, a combination of DTI and resting fMRI might provide the most promising early screening protocol to identify subjects “at risk” for developing ALS. However, further validation studies in larger patients' samples are required before these techniques can enter the clinical routine [7].
